# Molecular Alterations Associated with Breast Cancer Mortality

**DOI:** 10.1371/journal.pone.0046814

**Published:** 2012-10-04

**Authors:** Laura M. Voeghtly, Kim Mamula, J. Leigh Campbell, Craig D. Shriver, Rachel E. Ellsworth

**Affiliations:** 1 Clinical Breast Care Project, Windber Research Institute, Windber, Pennsylvania, United States of America; 2 Clinical Breast Care Project, Henry M. Jackson Foundation for the Advancement of Military Medicine, Windber, Pennsylvania, United States of America; 3 Clinical Breast Care Project, Walter Reed National Military Medical Center, Washington, D.C., United States of America; The Chinese University of Hong Kong, Hong Kong

## Abstract

**Background:**

Breast cancer is a heterogeneous disease and patients with similar pathologies and treatments may have different clinical outcomes. Identification of molecular alterations associated with disease outcome may improve risk assessment and treatments for aggressive breast cancer.

**Methods:**

Allelic imbalance (AI) data was generated for 122 invasive breast tumors with known clinical outcome. Levels and patterns of AI were compared between patients who died of disease (DOD) and those with ≥5 years disease-free survival (DFS) using Student t-test and chi-square analysis with a significance value of P<0.05.

**Results:**

Levels of AI were significantly higher in tumors from the 31 DOD patients (28.6%) compared to the 91 DFS patients (20.1%). AI at chromosomes 7q31, 8p22, 13q14, 17p13.3, 17p13.1 and 22q12.3 was associated with DOD while AI at 16q22–q24 was associated with DFS. After multivariate analysis, AI at chromosome 8p22 remained an independent predictor of breast cancer mortality. The frequency of AI at chromosome 13q14 was significantly higher in patients who died ≥5 years compared to those who died <5 years from diagnosis.

**Conclusion:**

Tumors from DOD compared to DFS patients are marked by increased genomic instability and AI at chromosome 8p22 is significantly associated with breast cancer morality, independent of other clinicopathological factors. AI at chromosome 13q14 was associated with late (>5-years post-diagnosis) mortality but not with death from disease within five years, suggesting that patients with short- and long-term mortality may have distinct genetic diseases.

## Introduction

Although mortality rates from breast cancer have decreased since 1990, it is estimated that ∼40,000 women in the United States died from breast cancer in 2011 [1]. Pathological characteristics such as architecture, differentiation, size and presence of local or distant metastasis, estrogen receptor (ER), progesterone receptor (PR) and HER2 expression are all considered when predicting prognosis and determining the most effective treatment options. At the molecular level, breast tumors have additional levels of variability, with at least five breast subtypes based on patterns of differential gene expression [2], [3]. This pathological and molecular heterogeneity influences prognosis and treatment: in a study of 2,929 breast cancer patients, despite use of identical treatment modalities for patients with similar pathological characteristics, clinical outcomes were highly variable [4].

This complexity suggests that the current standard of care approach to medicine, which is based on averaging responses across large cohorts, is insufficient to manage breast cancer. In contrast, personalized medicine provides care and treatment based on the individual characteristics of the tumor. For example, molecular profiling has improved pathological classification of tumors; molecular tests such as MammaPrint® and Onco*type* DX™ can provide estimates of the odds of recurrence and be used to discriminate those patients requiring aggressive treatment from those who would not benefit from cytotoxic regimens [5]–[7]. These RNA-based signatures are, however, not 100% accurate and can be affected by confounding factors such as RNA integrity and contamination of the tumor signature by stroma [8].

Although DNA exerts its effects at the RNA and/or protein levels, DNA is more stable than RNA and genomic alterations are not transiently affected by physiological conditions, thus identification of copy number alterations in primary breast tumor provides an alternate method to assess the underlying genetic composition of tumors with different phenotypes and behaviors. Identification of patterns of chromosomal changes associated with breast cancer mortality may be used to augment the predictive ability of currently available RNA and/or protein-based assays. In addition, identification of chromosomal alterations associated with aggressive tumor behavior should improve understanding of the biological changes associated with breast cancer mortality and may facilitate the development of new molecular therapeutics. Thus, to identify chromosomal alterations associated with poor prognosis, we subjected primary breast tumors from patients who died of disease (DOD) or were disease-free (DFS) >5 years post-diagnosis to allelic imbalance (AI) analysis. Data from DOD patients was further stratified by whether patients had short- (DOD within five years of diagnosis) or long-term (DOD > five years post-diagnosis) mortality to determine whether short- and long-term mortality has different molecular characteristics.

## Methods

### Ethics Statement

Tissue and blood samples from female Clinical Breast Cancer Project (CBCP) patients were collected with approval from the Walter Reed Army Medical Center Human Use Committee and Institutional Review Board. All subjects enrolled in the CBCP voluntarily agreed to participate and gave written informed consent. Clinical information was collected for all CBCP samples using questionnaires designed by and administered under the auspices of the CBCP.

### Materials

Paraffin-embedded primary breast tumors from patients diagnosed 2001–2006 were obtained from the CBCP Pathology Laboratory at Walter Reed Army Medical Center (WRAMC). Only primary tumors representing the original breast cancer diagnosis were subjected to AI analysis; tumors from patients who underwent neoadjuvant treatment were not included in this study. To ensure consistency, diagnosis of all tumor samples were made by one pathologist from hemotoxylin and eosin (H&E) stained slides; staging was performed using guidelines defined by the AJCC *Cancer Staging Manual* seventh edition [9] and grade was assigned using the Bloom-Richardson system of classification [10], [11]. Estrogen receptor (ER), progesterone receptor (PR) and HER2 status were determined by immunohistochemical (IHC) analysis (MDR Global, Windber, PA); cases with HER2 scores = 2+ were then evaluated by fluorescence in situ hybridization using the PathVysion® HER-2 DNA Probe kit (Abbott Laboratories, Abbott Park, IL).

### Methods

DNA was obtained from homogeneous populations of primary breast tumor cells following laser-assisted microdissection on an AS*LMD* laser microdissection system (Leica Microsystems, Wetzlar, Germany) [12]. All microdissected sections were examined by the CBCP pathologist, who identified and marked regions of tumor before dissection. The integrity of multiple serial sections was established by pathological verification of the first and last sections stained with H&E. Referent DNA (to determine the normal allelic intensities) samples were obtained from blood clots from each patient using Clotspin and Puregene DNA purification kits (Qiagen, Valencia, CA).

Microsatellite markers were amplified as previously described [13]. Genotypes were determined using Genetic Profiler version 2.0 software and AI detected using the formula (T1/T2)/(N1/N2) where T1 and N1 represent the peak heights of the less intense alleles and T2 and N2 represent the peak heights of the more intense alleles of the tumor and referent samples, respectively [14]. Each chromosomal region was represented by two polymorphic markers, and AI was defined according to the following criteria: (1) when at least one marker for a given region showed an allelic ratio ≤0.35, the region was considered to show AI, (2) when neither marker had an allelic ratio ≤0.35 and at least one marker was informative, the region was considered normal, and (3) when both markers were homozygous, the region was considered uninformative.

Comparison of the clinicopathological factors between groups and between AI levels and each factor was performed using chi-square analysis. Comparison of survival curves was determined using a log-rank (Mantel-Cox) test (GraphPad Prism™ v. 5.04). Nominal logistics regression analysis was used to measure the possible confounding effects of age at diagnosis, ethnicity, stage, grade, hormone receptor, lymph node and HER2 status, on the association between AI frequency and patient outcome. A significance value of *P*<0.05 was used for all analyses.

## Results

### Clinicopathological Characteristics

Of the 122 breast tumors assayed, 31 were from patients who DOD: 25 died within five years of diagnosis while six died more than five years post-diagnosis. Within the 91 DFS patients, four died of other causes more than five years post-diagnosis. When comparing clinicopathological factors between DOD and DFS patients, ethnicity, histological type and HER2 status did not differ significantly, however, tumors in DOD patients were diagnosed at a younger age (average 53.3 years in DOD and 59.2 years in DFS patients) and were significantly larger, of higher stage and grade, and more likely to have positive lymph node status and to be hormone receptor negative or of the triple negative subtype (Table 1).

**Table 1:** Clinical and pathological features of 123 invasive breast tumor specimens.

	DFS (n = 91)	DOD (n = 31)	P-value (DFS vs. DOD)
***Age at diagnosis***			**P<0.005**
<40 years	4%	13%	
40–49 years	26%	32%	
≥50 years	70%	55%	
***Ethnicity***			NS
White	65%	68%	
African American	24%	32%	
Asian	5%	0%	
Hispanic	6%	0%	
***Histology***			
IDCA	69%	90%	NS
ILCA	15%	6%	
Mixed	5%	4%	
Other^a^	11%	0%	
***Tumor size***			**P<0.05**
T1	68%	38%	
T2	24%	52%	
T3	8%	10%	
***TNM Stage***			**P<0.001**
I	53%	10%	
II	29%	19%	
III	16%	23%	
IV	2%	48%	
***Tumor Grade***			**P<0.005**
Low	37%	6%	
Moderate	28%	29%	
High	35%	65%	
***Lymph Node Status***			**P<0.001**
Negative	65%	13%	
Positive	35%	87%	
***Hormone Receptor Status***			**P<0.01**
ER+/PR+	61%	39%	
ER+/PR-	17%	10%	
ER−/PR-	22%	51%	
***HER2 Status***			NS
Positive	21%	23%	
Negative	79%	77%	
***Subtype*** b			**P<0.05**
Luminal A	67%	42%	
Luminal B	10%	6%	
HER2 enriched	7%	10%	
Triple Negative	16%	42%	

P-values listed as NS (not significant) >0.05.

a Other included histological types including tubular, medullary, apocrine, and mucinous carcinomas.

b IHC markers were used as surrogates to define subtype as follows: luminal A = ER and/or PR+/HER2−; luminal B = ER and/or PR+/HER2+; HER2-enriched = ER and PR−/HER2+ and triple negative = ER, PR and HER2−.

AI levels were compared for age at diagnosis, ethnicity, tumor size, stage, grade, lymph node, hormone receptor and HER2 status and intrinsic subtypes. Given the predominance of IDCA tumors compared to ILCA, mixed or other types, analysis by histology was not performed. In addition, given the small sample numbers for patients under 40 years, of Asian, Hispanic or other ethnicities, or with luminal B and HER2-enriched subtypes, age at diagnosis was restricted to patients <50 years compared to ≥50 years, only African American and Caucasian women were included when evaluating ethnicity, and luminal A tumors were compared to triple negative tumors. No significant differences were detected for age at diagnosis, ethnicity, hormone receptor or HER2 status or for intrinsic subtype. T2 tumors were found to have significantly higher levels of AI at chromosome 11p15 compared to T1, although none of the T3 tumors had detectable AI at this region. When comparing AI frequencies in early-stage (I/II) to late-stage (III/IV) tumors, chromosome 6q22.1–q23.1 was altered at significantly higher levels in late-stage compared to early-stage tumors, while chromosome 16q22–q24 was altered at significantly lower levels in late-stage compared to early-stage tumors. High-grade tumors had significantly higher levels of AI at chromosomes 7q31 and 17p13.1 compared to low- and intermediate-grade tumors. Lymph node positive tumors had higher AI at chromosomes 6q22–q23, 6q25–q27 and 17p13.1.

### AI in DOD and DFS Tumors

**Figure 1:**
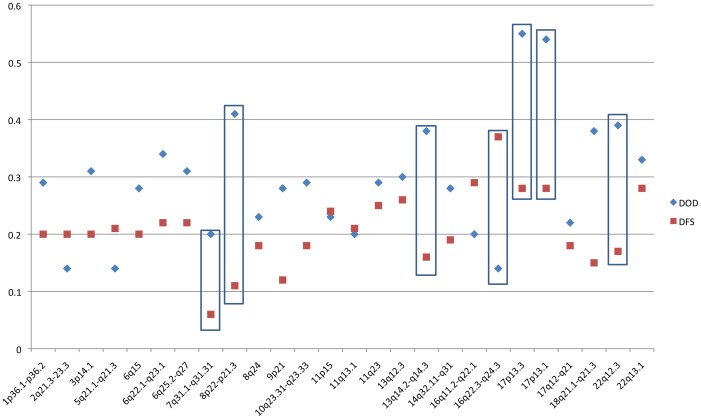
Scatter plot of levels of AI by chromosomal region. Chromosomal regions are on the x-axis, average level of AI on the y-axis. Chromosomes with significantly different frequencies between DOD and DFS groups are boxed.

Overall levels of AI were significantly higher in DOD (29%) compared to DFS (20%) tumors. Levels of AI varied by chromosome and patient group, with a low of 6% (5/82 informative DFS patients) at chromosome 7q31 to a high of 55% (12/22 informative DOD patients) on chromosome 17p13.3 (Figure 1). Multivariable analysis demonstrated that the association between high levels of AI and DOD remains significant, independent of other clinicopathological factors.

**Figure 2:**
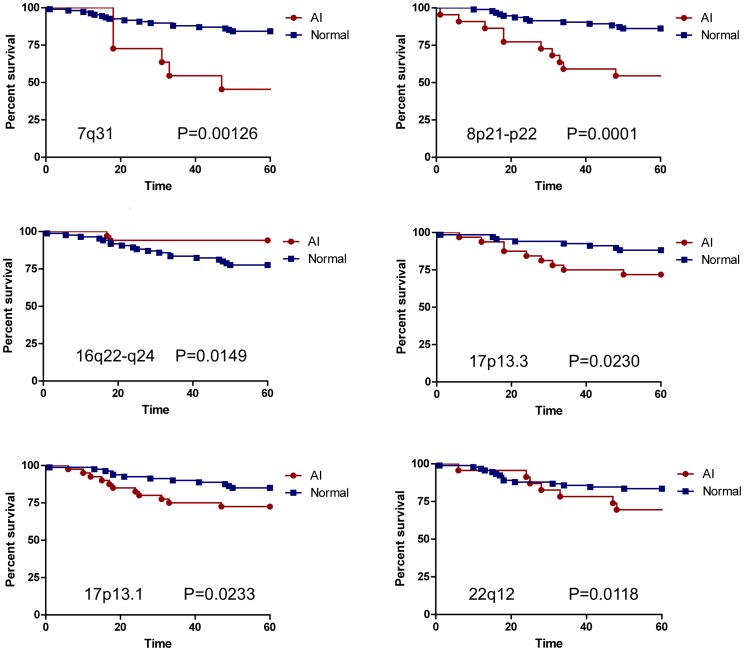
Survival curves of six chromosomal regions associated with significant differences in survival between patients with and without AI. Red circles = tumors demonstrating AI, blue squares = tumors without detectable AI. For chromosome 16q22–q24, those patients with AI at chromosome 16q22–q24 have significantly better survival than those with retention of chromosome 16q22–q24.

Survival curves were generated for each chromosome region. Survival analysis through 60 months supported a significant association between AI at regions 7q31, 8p22, 17p13.3, 17p13.1 and 22q12.3 and decreased survival (Figure 2). Conversely, AI of chromosome 16q22–q24 was associated with improved survival as patients who retained that region of chromosome 16q22–q24 had less favorable outcomes while those who had detectable AI had longer survival.

**Figure 3:**
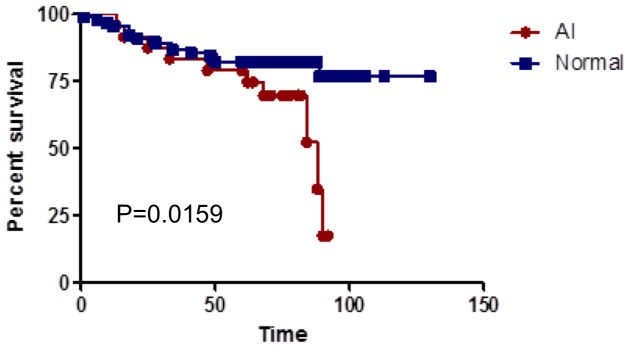
Extended survival curves (>60 months) for patients with AI at chromosome 13q14. The survival curves between patients with and without AI begin to separate only after 60 months (P = 0.0159), suggesting that alteration of 13q14 is associated with long-term mortality.

When survival was analyzed through five-years, no significant survival differences were detected for patients with AI at chromosome 13q14, however, when the analysis was extended, significant differences in survival were detected (Figure 3). These differences are largely accounted for by those patients who died of breast cancer more than 5 years post-diagnosis who had a significantly higher frequency of AI at chromosome 13q14 (83%) compared to those who died of disease within five years (25%) or the DFS (16%) patients. In contrast, AI of chromosome 16q22–24, which is associated with more favorable outcomes, was detected in 50% of patients who died after five years but in only one patient (5%) that died within five years of diagnosis.

To determine whether any of these regions was an independent predictor of outcome, multivariable analysis, including age at diagnosis, ethnicity, tumor stage, size and grade, and hormone receptor, HER2 and lymph node status, was performed to determine the relationship between AI and patient status. Six of the seven regions associated with outcome using survival analysis were no longer significant when other variables were measured; however, AI at chromosome 8p22 remained a predictor of status, independent of the effect of other pathological variables.

## Discussion

Although breast cancer treatment regimens are based largely on tumor characteristics such as tumor size and grade, ER, PR and HER2 status, traditional pathological evaluation is imperfect in predicting outcome [4]. Molecular characteristics may be more accurate prognostic tools than histological evaluation alone. For example, identification of intrinsic subtypes based on gene expression data separates breast tumors into different groups that have different biological characteristics, behaviors, rates of recurrence and sites of metastasis. Yet even within these subtypes, patients will have different outcomes [15]. Thus, it is important to determine whether tumors with aggressive behavior share common genetic alterations, regardless of phenotype.

Within our dataset, six chromosomal regions were altered at significantly higher frequencies in tumors from patients who died of disease, despite these tumors representing a range of pathological and molecular subtypes. Chromosomal alterations in a number of these regions have been previously associated with decreased survival in breast cancer patients, including AI of chromosomes 7q31, 8p22, and 17p13.3, and copy number alterations of 8p, and 13q [15]–[18].

In addition to the six regions with significantly higher levels of AI in tumors from DOD patients, retention of chromosome 16q22–q24 was associated with breast cancer mortality. Chromosomal alterations of 16q22–q24 have been associated with favorable tumor characteristics such as low-grade, higher age at diagnosis, positive ER status and the luminal A subtype, which has the most favorable prognosis [19]–[21]. Loss of chromosome 16q22 and decreased expression of genes from chromosome 16q22 and 16q24 have also been associated with improved survival [22], [23]. These data suggest metastasis promoting genes exist within these regions; loss or AI at these regions such as seen at significantly higher levels in DFS (37%) and DOD patients who died >5 years diagnosis (50%), would therefore be protective, while in DOD patients who died within five-years (10%), retention of this region may promote early DOD.

Multivariate analysis has demonstrated that many of these regions associated with poor outcome are also associated with other clinicopathological factors. For example, while AI at 17p13.1 is associated with decreased survival (P = 0.0233), when analyzed in a multivariable model, tumor grade exerts a significant influence. This result is supported by earlier studies from our laboratory, both of which included a subset of the samples reported here: evaluation of tumor grade found that 17p13.1 was altered in a significantly higher proportion of high-grade compared to low-grade tumors [20], and in a follow-up study evaluating the effect of chromosomal alterations on tubule formation, nuclear atypia and mitosis, AI at chromosome 17p13.1 was associated with high levels of nuclear atypia [24]. In addition, while AI at chromosome 16q22–q24 was associated with 5-year DFS, the effect of AI at chromosome 16q22–q24 on outcome was mitigated in a multivariable model by a number of factors including HER2 status and grade. We have previously found associations between AI at 16q22–q24 and low-grade tumors and HER2 amplification [20], [25].

Forty-one percent of DOD patients had detectable AI at 8p22 compared to just 11% of DFS patients. The region of 8p22 assayed here represents a 1.9 MB flanked by microsatellite markers D8S552 and D8S511 [13]. Loss of this region of chromosome 8p has been associated with large tumor size and aggressive histology, increased proliferation, increased five-year post-operative mortality, increased recurrence in early-stage breast tumors, decreased survival in young women and metastasis [17], [23], [26]–[31]. This region of chromosome 8p harbors four genes: KIAA1456, deleted in liver cancer 1 (DLC1), C8orf48 and sarcoglycan, zeta (SGCZ). KIAA1456 and C8orf48 are not well-characterized; SGCZ encodes a N-glycosylated transmembrane protein which provides a link between the cytoskeleton and extracellular matrix. A link between SGCZ, KIAA1456 or C8orf48 and breast cancer has not been established, however, DLC1 has been shown to inhibit cancer cell growth and been implicated as a tumor suppressor [32], [33]. In a mouse model of hepatocellular cancer expression of DLC1 significantly suppresses dissemination of tumor cells, while restoration of DLC1 expression in a mouse breast cancer model resulted in a significant decrease in metastases [34], [35]. Together, these data suggest the DLC1 may play an important role in the development of distant metastasis and mortality of breast cancer patients.

The genetic differences between patients who were DOD within five years diagnosis and those that died > five years may provide a model for early and late breast cancer mortality. The two groups of patients had similar overall levels and patterns of AI except at chromosomes 13q14 and 16q22–q24. A high frequency of AI at chromosome 13q14 was detected in 83% of late-term DOD patients but only 25% of short-term DOD and 16% of DFS patients. Dysfunction of the retinoblastoma (RB1) gene, which maps to chromosome 13q14, has been associated with increased risk for cancer-specific death, in patients who have survived at least five years from diagnosis [36]. Of the 14 DFS patients who had AI at chromosome 13q14, one died of other causes. The remaining 13 patients have been alive an average of 76 months (range 60–92 months) since diagnosis, however, given the presence of AI at chromosome 13q14, these patients may be at increased risk for cancer mortality.

In conclusion, tumors from patients who died from breast cancer differ at the chromosomal level from patients with good prognosis. The higher frequency of AI in DOD tumors suggests that increased genomic instability is associated with poor prognosis. In addition, AI at chromosome 8p22 in the DLC1 gene region is associated with breast cancer mortality, independent of other pathological factors while AI at chromosome 13q14 is associated specifically with late mortality. These data improve our understanding of the molecular underpinnings of breast cancer mortality and suggest patients with short- and long-term mortality may have distinct genetic diseases.
